# Child maltreatment affects fathers’ response to infant crying, not mediated by cortisol or testosterone

**DOI:** 10.1016/j.cpnec.2021.100083

**Published:** 2021-08-28

**Authors:** Martine.W.F.T. Verhees, Marinus H. van IJzendoorn, Kim Alyousefi-van Dijk, Anna M. Lotz, Noor de Waal, Marian J. Bakermans-Kranenburg

**Affiliations:** a Clinical Child & Family Studies, Vrije Universiteit Amsterdam, the Netherlands; b Department of Psychology, Education and Child Studies, Erasmus University, Rotterdam, the Netherlands

**Keywords:** Fathers, Childhood maltreatment, Hair cortisol, Hair testosterone, Parenting

## Abstract

Parents' ability to appropriately respond to infant crying is essential for parental care and has been found to relate to parents' own childhood experiences. Additionally, childhood experiences can affect endocrine factors, which may subsequently affect behavior. In the current study, preregistered on https://osf.io/hwgtu, we examined in expectant and new fathers (*N* = 152) associations between experiences of maltreatment in their own childhood, hair cortisol and testosterone concentrations and their ability to modulate handgrip force when exposed to infant crying. Cortisol and testosterone were quantified from the 1 cm of hair most proximal to the scalp using Liquid chromatography-tandem mass spectrometry. Participants were asked to squeeze a handgrip dynamometer at full and half strength while listening to infant cries and control sounds. Results indicated that fathers who experienced more childhood maltreatment used more excessive handgrip force during infant cry sounds. Hair cortisol and testosterone were not related to either experienced childhood maltreatment or handgrip strength modulation. These findings confirm that fathers’ early experiences of maltreatment reduce their ability to modulate their behavioral responses during infant cries, but suggest that hair cortisol and testosterone concentrations do not identify the underlying mechanism of this association.

## Highlights

• Childhood maltreatment relates to fathers' behavioural responses to infant crying.
• Childhood maltreatment is unrelated to fathers' hair cortisol and testosterone.
• Cortisol and testosterone are unrelated to behavioural responses to infant cries.

## 1 Introduction

Infant crying is an important signal indicating infant distress and parents’ ability to appropriately respond to crying is essential for parental care [1]. However, cries can be perceived as aversive and prompt harsh parenting [35]. Both maltreating mothers and parents at risk for perpetrating maltreating behavior have a reduced ability to modulate their behavioral responses, such as handgrip force, while hearing infant cries [16,18]. Therefore, the inability to modulate handgrip force when exposed to infant crying can be considered a risk factor for harsh parenting behavior. It is crucial to examine factors and mechanisms involved in responses to infant crying to elucidate why some parents are more at risk to respond harshly. This may not only advance our theoretical understanding of harsh parenting behavior, but may ultimately also help to reveal opportunities for prevention and intervention efforts to reduce harsh parenting and associated negative child outcomes (e.g., [39,56]).

**Fig. 1:**
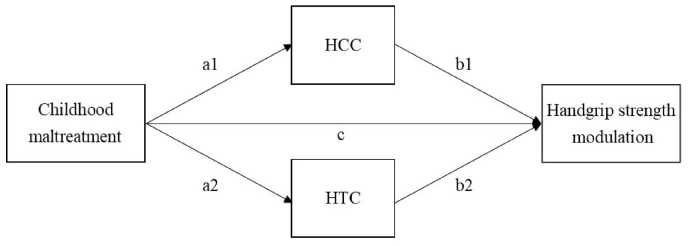
Overview of the research questions. HCC = Hair Cortisol Concentration; HTC = Hair Testosterone Concentration.

This study, preregistered on https://osf.io/hwgtu, focused on expectant and new fathers, an often neglected group in parenting research. In one of our previous studies we showed that fathers’ own childhood experiences are relevant for their reactions to infant cries, as fathers with more childhood maltreatment experiences tend to use excessive handgrip force during exposure to infant crying [2] (and see [13] for similar findings in a different sample including mothers and fathers). In the current study on a larger sample including the earlier one, we aimed to confirm and extend this finding and examined whether (1) experienced childhood maltreatment positively relates to paternal use of excessive handgrip force during infant crying, (2) experienced maltreatment is associated with hair cortisol concentration (HCC) and hair testosterone concentration (HTC) in adulthood, and (3) HCC and HTC in turn relate to the use of excessive handgrip force. We additionally tested (4) indirect effects of experienced maltreatment on handgrip strength modulation via HCC and HTC (see Fig. 1).

Early experiences can affect the organization of hormone receptors or hormone production and secretion [19]. Experiencing childhood maltreatment has been associated with Hypothalamic-Pituitary-Adrenal axis dysregulation, resulting in altered basal or reactive cortisol levels [52]. Findings on the association between maltreatment and cortisol levels are mixed. Two meta-analyses reported reduced salivary cortisol responses to stress [14] and wake-up cortisol levels [9]. Another meta-analysis focused on HCC [28], providing a longer-term measure of cortisol secretion compared to salivary measures reflecting acutely circulating cortisol. The latter meta-analysis revealed both elevated and blunted HCC in individuals who experienced adverse life events, including childhood maltreatment, with an overall combined effect size of *d* = 0.21, pointing to elevated cumulative cortisol which indicates long-term physiological stress. Based on this finding we hypothesized that fathers’ experienced childhood maltreatment is associated with elevated HCC (Fig. 1, a1-path).

Moreover, although there is a lack of empirical research on the effect of childhood maltreatment experiences on basal testosterone levels in adulthood, life history theories propose that experiencing early life stress or harsh parenting relates to accelerated pubertal development which may reflect (possibly temporarily) heightened levels of gonadal hormones such as testosterone [8,11,20]. To our knowledge, only one study tested whether childhood experiences are associated with testosterone in adults and found no association [57], but this study examined testosterone reactivity to caregiving in women rather than basal testosterone levels in men. Based on life history theories, we tentatively hypothesized to find a positive association between fathers’ experienced childhood maltreatment and HTC (Fig. 1, a2-path).

Recently, there has been increasing attention to endocrine factors playing a role in parents' behavioral responses to infant signals [4]. Maternal plasma and salivary cortisol levels have been positively related to affectionate infant-directed behavior and sympathy towards infant cries in the first postpartum days [23,49]. In fathers, salivary cortisol sampled on the day of birth positively predicted paternal involvement 2–4 months postpartum [31]. However, these effects may be specific to cortisol measured very early postnatally as maternal sensitivity has been negatively associated with salivary cortisol at 2–6 months postpartum [27] and across the first two postpartum years [21]. One study in fathers found that higher prenatal salivary cortisol was related to lower postnatal sensitivity observed from fathers' interaction with their own infant, but not to their prenatal sensitivity as observed with an infant simulator [12]. Whereas the majority of studies in the context of parenting measured hormones in saliva, here we used HCC and HTC, retrospectively capturing hormone levels in the month prior to hair sampling. HCC has been found to significantly correlate with total salivary cortisol secretion over a longer-period of time (e.g. [47,51]), suggesting that HCC is suitable to measure longer-term baseline cortisol levels. Persistent high levels of stress, as reflected by enhanced HCC levels, interfere with parents’ ability to appropriately respond to infant signals as perception, cognition, and action systems related to these responses are compromised by chronic stress [7,53]. We therefore hypothesized that higher levels of HCC would relate to more excessive handgrip force use during infant crying (Fig. 1, b1-path).

Regarding testosterone, a meta-analysis revealed a negative association of basal testosterone levels with paternal caregiving quality, albeit with a small effect size [33]. Fathers with higher basal salivary testosterone levels show more neural activation to infant cries which may indicate hyperreactivity [29]. They seem less sympathetic to infant cries and feel less need to respond to them [22], although increases in salivary testosterone may also signal a readiness to respond to infant crying [55]. High testosterone has been associated with low impulse control and higher levels of aggression in general (e.g., [[59], [60]]), suggesting that parents with higher testosterone levels may tend to respond more harshly to infant signals. In line with this suggestion, high paternal testosterone levels have been related to elevated risk for child maltreatment [42]. To our knowledge, no previous studies assessed the association between long-term testosterone levels as measured with hair and (indicators of) parenting behavior. Nevertheless, based on the literature cited above, we hypothesized that HTC would positively relate to paternal use of excessive handgrip force (Fig. 1, b2-path). Following the dual hormone hypothesis [32], stating that testosterone effects on behavior depend on an individual's cortisol levels, and previously reported interactive effects of salivary cortisol and testosterone on parenting [12], we explored in a post-hoc analysis whether HCC and HTC interacted in their effect on handgrip strength modulation.

As cortisol and testosterone may relate to both early experiences and parenting, they may mediate the association between experienced childhood maltreatment and handgrip strength modulation [27,30,34]. To our knowledge, no studies assessed these associations in fathers. Here we tested whether HCC and HTC mediate the association between fathers’ experienced childhood maltreatment and handgrip strength modulation during infant cry (Fig. 1, ab-paths).

As evident from the discussed literature, with the current research questions concerning HCC and HTC, we cannot rely on a solid base of previous work addressing the same questions in fathers. This study may therefore be seen as an exploratory study that aims to provide benchmarks and priors for future research on the interplay between childhood experiences, hormones and parenting in fathers.

## 2 Material and methods

### 2.1 Participants

**Fig. 2:**
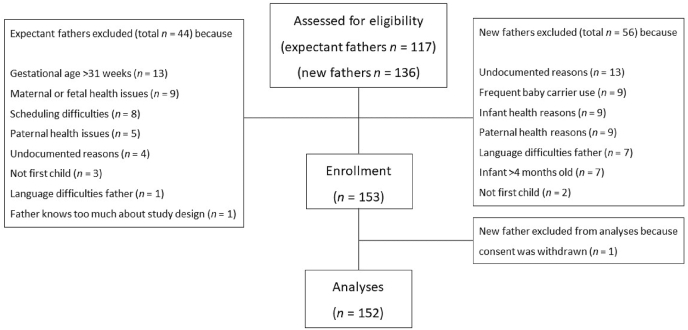
Flowchart of the inclusion in the study.

First-time expectant and new fathers were recruited via midwife practices, child healthcare centers, municipal records and (online) advertisements. A total of 152 fathers were included in the current study (73 expectant fathers, 79 new fathers; see Fig. 2 for a flowchart and Supplementary material S1 for a priori stated inclusion and exclusion criteria). Due to recruiting difficulties, we deviated from preregistered exclusion criteria in 20 cases (12 MRI contraindications, these participants were included but did not undergo MRI scanning; one participant had a cardiovascular disease; three participants had ADHD; one participant had diabetes and used medication potentially interfering with the endocrine system (metformin); two participants used medication potentially interfering with the endocrine system (rhinocort nasal spray and venlafaxine); one child was born after 36 weeks and 6 days of gestation, but was considered healthy).

Fathers were 25–56 years old (*M* = 32.87, *SD* = 4.47), were mainly born in the Netherlands (93%) and followed on average 8.53 years of education following primary school (*SD* = 1.68). Expectant and new fathers did not differ in age or education, *t*s < 1.91, *p*s > .05, or country of birth (Netherlands vs other), *χ*^*2*^ (1) = 0.86, *p* = .35. Infants of expectant fathers had a mean gestational age of 24.95 weeks (*SD* = 2.82) and 49% of these infants were female (32% were male, for 19% sex was unknown at time of assessment). Infants of new fathers were on average 11.62 weeks old (*SD* = 3.37) and approximately half of them were male (53%). One new father was not the biological father of the child, but had been living with the biological mother since mid-pregnancy.

### 2.2 Measures

#### 2.2.1 Experienced childhood maltreatment

Experienced childhood maltreatment was measured using two questionnaires. The Parent Child Conflict Tactics Scale (CTSPC) [50] assessed participants' experienced childhood maltreatment by their parents. Eighteen items were rated for mother or father (the parent for whom the highest score was applicable) on a 7-point scale (0 = ‘never’, 1 = ‘once’, 2 = ‘twice’, 3 = ‘3–5 times’, 4 = ‘6–10 times’, 5 = ‘11–20 times’, 6 = ‘more than 20 times’). We included the subscales Psychological aggression (5 items), Physical assault (8 items) and Neglect (5 items). Mean scores on the Psychological aggression and Physical assault scales were averaged to form an Abuse scale. To create a total CTSPC score, scores on the Abuse scale and mean scores on the Neglect subscale were averaged (*M* = 0.72, *SD* = 0.71, range = 0.00–3.48; *α* = 0.85 across all CTSPC items). Additionally, participants reported on their childhood experiences of parental love withdrawal in an 11-item questionnaire, which contained seven items of the Withdrawal of Relations subscale of the Children's Report of Parental Behavior Inventory (CRPBI) [10,46], and four items of the Parental Discipline Questionnaire (PDQ) [36]. The 11-item questionnaire has been used previously to measure parental love withdrawal in Dutch community samples (e.g., [54]). Participants indicated on a 5-point scale ranging from 1 (‘not at all’) to 5 (‘very well’) how well each statement described their parent's behavior, for father and mother separately (e.g., ‘My mother was a person who, when I disappointed her, told me how sad I made her’). To create a parental love withdrawal score, for each item the highest score was taken (being either reported about father or mother) and these highest scores were averaged across all 11 items (*M* = 2.04, *SD* = 0.81, range = 1.00–4.18; *α* = 0.90). We created a total childhood maltreatment score by averaging the standardized CTSPC score and standardized love withdrawal score (*α* = 0.89 across all items of both questionnaires). Two outliers (*z* > 3.29) were winsorized, i.e., transformed to comply with the distribution while keeping the rank order within the distribution. There was no difference between expectant and new fathers on experienced childhood maltreatment, *t*(144) = 1.71, *p* = .09.

#### 2.2.2 HCC and HTC

To determine participant's HCC and HTC, a strand of hair with a diameter of approximately 3–5 mm was cut from the inion, as close to the scalp as possible. Hair samples were stored at room temperature in aluminum foil before being shipped to Dresden Labservices GMBH for analysis. The 3 cm of hair most proximal to the scalp were cut into three segments of 1 cm each that were used for further analyses. HCC and HTC were quantified using Liquid chromatography-tandem mass spectrometry with online solid phase extraction (LCMS/MS) [25]. Inter-assay variability has been established as less than 9% for HCC and HTC [25]. A random selection of 31% of the hair segments were assayed in duplicate and intra-assay coefficients of variation were 11% and 14% for HCC and HTC, respectively. For segments assayed in duplicate, the mean value of the two assays was used for further analysis. HCC and HTC segments 1, 2 and 3 values were log-transformed as their distribution was positively skewed. There were one HCC segment 1 outlier, two HTC segment 1 outliers and one HTC segment 2 outlier (*z* < −3.29), which were winsorized. After log-transformation and outlier winsorizing, distribution of HTC segment 1 data was still skewed (*z* = −5.38). Therefore, for analyses with HTC segment 1 as outcome variable, we ran sensitivity analyses with bootstrapped standard errors (1000 samples). There were no differences between expectant and new fathers on HCC or HTC values for any of the segments, *t*s between −0.25 and 1.90, *p*s ≥ .06. In this study's main analyses, we used HCC and HTC values of the 1 cm segment most proximal to the scalp. These values had the lowest number of missings, given short hair length of some fathers, and reflect hormone secretion in the month prior to hair sampling, as hair grows approximately 1 cm per month [48].

#### 2.2.3 Handgrip strength modulation

A handgrip dynamometer paradigm was used to measure participants’ ability to modulate handgrip strength during infant crying. Test-retest reliability for handgrip strength measurement using a dynamometer has been shown adequate across a ten-week period [38]. Participants were asked to squeeze a handgrip dynamometer at full and half strength while listening to infant cry sounds and neutral control sounds. Participants first went through a training phase, during which they alternated between full and half-strength squeezes without being exposed to sounds. Squeeze intensities (in kg) were transferred directly from the dynamometer to AcqKnowledge software (version 4.3.1; Biopac Systems, 2004). Participants received feedback about their squeeze strength via a monitor and practiced until they were able to modulate the force of the second squeeze to half the strength of the first squeeze. Then the actual task started during which participants no longer received feedback on their squeeze intensity.

During the task, participants were seated in front of a computer screen while wearing headphones. The task was administered using E-prime software (version 2.0; Psychology Software Tools, Pittsburgh, PA, USA). Participants first performed three baseline trials (hearing no sound) of full-strength squeezes each followed by a half-strength squeeze. Then participants performed a total of 12 full-half-strength trials, six times while listening to infant crying and six times while listening to control sounds. Each trial lasted 12s. After 8s, participants were prompted to squeeze at full strength for 1s, followed 2s later by a half-strength prompt that was shown for 1s. In-between trials a fixation cross was shown for 3s. Infant cry sounds were recorded from six infants between two days and 5.5 months old. Cry sounds were scaled, their intensity was normalized to 74 dB and they were edited to last 10s using PRAAT. From each cry sound the average spectral density and amplitude modulated by the amplitude envelope was extracted and resynthesized in order to create a control sound that matched the cry stimulus on intensity, duration, spectral content and amplitude envelope, but lacked its emotional valence.

In addition to listening to the cry and control sounds, participants watched images that either resembled their own infant or another infant (for a description of how prenatal images were created, see [3]). This resulted in four conditions (own child neutral sound, own child cry sound, other child neutral sound, other child cry sound) of three full-half strength trials each that were presented to participants in a semi-randomized order.

Peak intensities for each squeeze were identified using Matlab (version 8.0.0.783; Mathworks, MA, USA). Grip strength modulation was calculated by dividing the half-strength squeeze intensity by the preceding full-strength squeeze intensity (see e.g., [16]). Scores over 0.50 thus reflected the use of excessive force on the half-strength squeeze attempt. Scores below zero or over two were considered measurement errors and were disregarded. Handgrip ratios were averaged per condition (*α*s between 0.74 and 0.86). For the current study, the own versus other child distinction was not of interest and mean handgrip ratios to cry and control sounds were averaged across the own and other child conditions (correlations across these conditions were high, *r* = .78 for cry sounds, *r* = .73 for control sounds). A cry-control sound contrast was created by residualizing the squeeze ratio during cry trials for the squeeze ratio during control sounds (see [2] for the same approach). This can be considered a measurement of reactive force during infant cries, controlled for force during neutral sounds. One outlier (*z* > 3.29) was winsorized. There was no difference between expectant and new fathers on residualized handgrip force scores, *t*(148) = 1.31, *p* = .19.

#### 2.2.4 Covariates

##### 2.2.4.1 Hair covariates

Previous research identified several potential covariates of HCC, i.e., age, hair washing frequency, hair treatment, body mass index (BMI), medication use, hair color, astronomical season, socio-economic status, and race [41,48]. Additionally, mass of the hair segment may be related to HCC (C. Kirschbaum, personal communication, May 4, 2020). Less is known about covariates of HTC, although age and BMI may be related to HTC [15]. We assessed these potential covariates to allow controlling for them in the analyses (see Supplementary material S2 for details on how covariates were measured).

##### 2.2.4.2 Perinatal depression

To allow controlling for current depressive symptoms, participants completed the Edinburgh Postnatal Depression Scale (EPDS) [17]. The EPDS contains 10 items concerning how the participant felt during the previous week (e.g., ‘I have looked forward with enjoyment to things’, *α* = 0.76). A sum score was calculated to reflect perinatal depression.

### 2.3 Procedure

Data was collected during research visits that took place at the lab (84% of the visits), at home (13%), or partly at the lab and partly at home (3%), dependent on whether participants underwent MRI scanning and on the participant's preference. The full procedure comprised a set of questionnaires, sampling of hair and saliva for determination of hormone levels, a 10-min period taking care of an infant simulator (for expectant fathers) or a 10-min father-infant free play period (for new fathers), a second sampling of saliva, MRI scans (only for lab visits), the handgrip dynamometer task, and 5 min during which fathers talked about their baby. After the session, participants received a set of online questionnaires to complete at home, which included the childhood maltreatment questionnaires. As part of the full study procedures fathers were tested two additional times, but data from these follow-up sessions was not used here. The study procedures were approved by the medical ethics committee of the Leiden University Medical Centre and the ethics committee of the Department of Education and Child studies at Leiden University. All participants provided written informed consent. Participants received a financial compensation of €25 (plus travel allowance) for their participation in the assessment described here and additionally received €10 if they completed at least 80% of the questionnaires at the end of the study.

### 2.4 Analyses

The current study was powered to detect medium effect sizes. A detailed power analysis can be found in the preregistration (https://osf.io/hwgtu).

#### 2.4.1 Missing data

Missingness rates per variable were as follows: 3.9% experienced maltreatment, 13.2% HCC segment 1, 43.4% HCC segment 2, 71.1% HCC segment 3, 29.6% HTC segment 1, 54.6% HTC segment 2, 73.7% HTC segment 3, 1.3% handgrip strength modulation, 3.9% EPDS, 0.7% educational level father, 10.5% hair color, 12.5% hair mass segment 1, 42.8% hair mass segment 2, 69.7% hair mass segment 3, 0% for all other hair covariates. For HCC, HTC and hair mass of segment 2 and 3, missingness rates were high as many participants' hair was shorter than 2 or 3 cm. Little's missing completely at random (MCAR) test indicated that data was MCAR, *Χ*^*2*^(444) = 475.10, *p* = .15. Full information maximum likelihood (FIML) was used to handle missing data in the main analyses.

#### 2.4.2 Hair covariates

We tested on full-cases data whether there were significant associations between the potential hair covariates and HCC and HTC segment 1 values. For continuous covariates (age, BMI, educational level, hair segment mass), Pearson correlations revealed no significant correlations with HCC segment 1 (*p*s > .83) or HTC segment 1 (*p*s > .28). For categorical covariates (hair washing frequency, hair washed past 24h, hair product use frequency, hair product use past 24h, medication use past 48h, hair color, race, astronomical season), ANOVAs revealed that participants who had washed their hair in the past 24 h had lower HCC values compared to participants who had not, *F*(1,130) = 4.26, *p* = .04. Moreover, astronomical season of the lab visit related to both HCC, *F*(3,128) = 10.93, *p* < .001, and HTC, *F*(3,103) = 2.77, *p* < .05. Fathers who participated in summer had higher HCC than those who participated in any other season, and had higher HTC than those who participated in fall. There were no other significant associations between categorical covariates and HCC or HTC segment 1 (*p*s > .06). HCC values were corrected for hair washed in the past 24h and season (summer vs other) and HTC values were corrected for season (summer vs other) via residualizing.

#### 2.4.3 Main analyses

All main analyses were performed in R using the package lavaan [44], using the maximum likelihood (ML) estimator. To test the association between experienced childhood maltreatment and handgrip strength modulation, we performed a linear regression analysis with experienced maltreatment as predictor and handgrip strength modulation as outcome variable. To test the association between experienced maltreatment and HCC, and HTC, we performed two linear regression analyses, both with experienced maltreatment as predictor, one with HCC as outcome variable and one with HTC as outcome variable. To test the relation between HCC and HTC, and handgrip strength modulation, we performed two linear regression analyses, one with HCC as predictor and one with HTC as predictor, both with handgrip strength modulation as outcome. For non-significant main effects, we calculated Bayes factors that quantify the relative evidence in the data in favour of the hypothesis that there were, at least, small effects (β > |0.10|) against the hypothesis that there were no effects (β = 0), using the program bain in R [24]. Finally, to simultaneously test the effects of experienced childhood maltreatment on handgrip strength modulation, HCC and HTC, and the effects of HCC and HTC on handgrip strength modulation we specified a structural model. We tested indirect effects between experienced maltreatment and handgrip strength modulation via HCC and HTC. Standard errors were bootstrapped (1000 samples).

#### 2.4.4 Sensitivity and exploratory analyses

We conducted a set of a preregistered sensitivity analyses. First, as current depressive symptoms may (in part) account for obtained results, we reran the analyses controlling for depressive symptoms as measured with the EPDS. Second, we ran sensitivity analyses excluding participants using medication that potentially interferes with the endocrine system. Third, we ran the mediation analysis for HCC and HTC separately to allow for testing whether HCC suppressed the effects of HTC and vice versa. Fourth, we examined whether effects held when even longer-term hormone secretion was included in the mediation model. Specifically, we added an equation part to the model and included two latent factors, for HCC and HTC respectively, based on all three 1 cm hair segments. These latent factors reflect the hormone secretion in the three months prior to assessment. We included the latent factors in a sensitivity analysis and not in the main analyses for two reasons: adding an equation part to the model decreases statistical power, and many participants had missings for the second and third cm of hair because their hair was too short.

In a post-hoc exploratory analysis, we examined whether HCC and HTC interacted in their effects on handgrip strength modulation. To this aim, we performed a regression analysis with handgrip strength modulation as outcome variable and mean-centered HCC, HTC and their interaction term as predictors. In addition, we explored whether there were differences between new and expectant fathers by performing a multi-group mediation analysis.

## 3 Results

### 3.1 Descriptive statistics

Descriptive statistics for the main variables based on full-cases are presented in Table 1.

**Table 1:** Descriptive statistics for the main variables.

Variable	N	Mean (SD)	Range
Maltreatment	146	−0.01 (0.84)	−1.15 – 2.39
HCC			
Segment 1	132	0.61 (0.32)	−0.08 – 1.51
Segment 2	86	0.63 (0.33)	0.05–1.67
Segment 3	44	0.61 (0.32)	−0.02 – 1.24
HTC			
Segment 1	107	−0.34 (0.44)	−1.76 – 0.59
Segment 2	69	−0.09 (0.38)	−0.99 – 0.91
Segment 3	40	0.07 (0.36)	0.47–0.98
Handgrip strength modulation	150	−0.01 (0.97)	−3.11 – 2.69

*Note.* Maltreatment is the average of the standardized scores on the Parent Child Conflict Tactics Scale and the love withdrawal scale. HCC = Hair cortisol concentration (pg/ml), log 10; HTC = Hair testosterone concentration (pg/ml), log10. Handgrip strength modulation is the standardized residual from the squeeze ratio during cry trials predicted by the squeeze ratio during control sounds.

### 3.2 Main analyses

#### 3.2.1 Experienced maltreatment and handgrip strength modulation

There was a positive association between experienced maltreatment and handgrip strength modulation, β = 0.19, *p* = .02, *N* = 152. Fathers who experienced more childhood maltreatment used more excessive handgrip force during infant crying.

#### 3.2.2 Experienced maltreatment, and HCC and HTC

There was no relation between experienced maltreatment and HCC, β = 0.11, *p* = .24, *n* = 151. A Bayes factor indicated that the data provided 5.58 times more support for a null effect compared to at least a small effect. Additionally, experienced maltreatment was not associated with HTC, β = 0.03, *p* = .79, *n* = 150. Results with bootstrapped SEs (as HTC was not normally distributed) were similar. A Bayes factor showed that the data provided 26.30 times more support for a null effect compared to at least a small effect.

#### 3.2.3 HCC and HTC, and handgrip strength modulation

HCC was not associated with handgrip strength modulation, β = 0.10, *p* = .25, *N* = 152. A Bayes factor revealed 6.28 times more support in the data for a null effect compared to at least a small effect. HTC levels also did not relate to handgrip strength modulation, β = −0.09, *p* = .34, *n* = 151. A Bayes factor indicated that the data provided 8.18 times more support for a null effect compared to at least a small effect.

#### 3.2.4 Indirect effects between experienced maltreatment and handgrip strength modulation via HCC and HTC

**Fig. 3:**
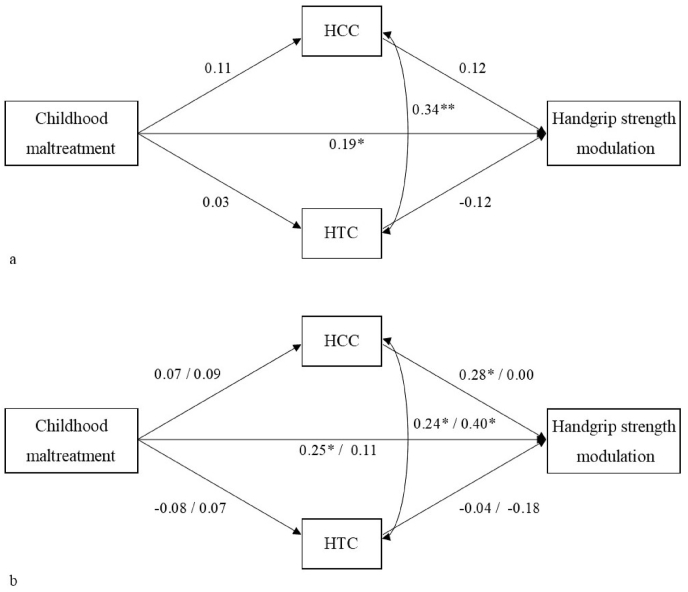
Structural models with standardized coefficients along the paths. 3a. Model for the full sample (N = 152). 3b. Models for the group of expectant (n = 73) vs new (n = 79) fathers. Coefficients for expectant fathers are presented left of the slash, coefficients for new fathers are presented right of the slash. HCC = Hair Cortisol Concentration; HTC = Hair Testosterone Concentration. *p <.05, **p < .01.

None of the direct paths to and from HCC and HTC were significant, however, for completeness and per preregistered analytic plan we tested the indirect effects via HCC and HTC. We allowed HCC and HTC to covary. The model is shown in Fig. 3a. Neither of the indirect effects was significant (*p*s > .51).

### 3.3 Sensitivity and exploratory analyses

First, we ran the analyses additionally controlling for current depressive symptoms, by including EPDS as a predictor in our models. Results remained similar in terms of direction and *p*-values.

Second, we excluded participants using medication that potentially interferes with the endocrine system. Three participants reported using such medication at time of inclusion. An additional nine participants reported at the day of assessment that they used medication potentially interfering with the endocrine system in the past 48h (e.g., medication containing corticosteroids). Excluding these 12 participants in the analyses did not affect the results in terms of significance or direction.

Third, we ran the mediation analysis for HCC and HTC separately. Indirect effects remained non-significant in the single mediation models (*p*s > .59).

Fourth, we examined whether effects held when latent factors reflecting hormone secretion in the past three months were included in the mediation model. Latent variable variances were fixed to 1. We allowed the latent factors HCC and HTC to correlate. Model fit was acceptable based on the comparative fit index (CFI = 0.93), and mediocre based on the root mean square error of approximation (RMSEA = 0.09). The latent factors HCC and HTC were positively associated, β = 0.43, *p* < .001. There were no significant direct paths from or to HCC and HTC (*ps >* .16) and the indirect effects via HCC and HTC were also non-significant (*p*s > .77).

Additionally, we ran an exploratory analysis to examine whether HCC and HTC interacted in their effect on handgrip strength modulation. The interaction-term was not associated with handgrip strength modulation, β = −0.02, *p* = .89, *N* = 152.

Finally, we ran a multi-group mediation analysis to explore whether there were differences between expectant and new fathers. The models for expectant and new fathers are presented in Fig. 3b. There were no significant differences between the two groups for any of the direct or indirect effects (*p*s > .11). The models showed that the path from maltreatment to handgrip strength modulation was only significant in the group of expectant fathers, but the effect sizes in the two groups were not significantly different. Moreover, in expectant fathers, HCC and handgrip strength modulation were positively associated, indicating that fathers with higher HCC used more excessive handgrip force during infant crying.

## 4 Discussion

This study tested associations between childhood maltreatment experiences, HCC and HTC levels, and handgrip strength modulation during infant crying in expectant and new fathers. As predicted, results showed that experienced childhood maltreatment related to fathers' increased use of excessive handgrip force during infant cries. This finding seems robust as it confirms earlier findings in a sample including mothers and fathers [13] and in a subsample of the current sample [2]. Individuals who have experienced childhood maltreatment show increased attention to negative stimuli and tend to interpret stimuli more negatively [26]. Moreover, experiencing childhood maltreatment has been related to an impaired ability to regulate and tolerate negative emotions [61]. Infant cries may trigger more aversion, negative arousal and frustration in fathers with maltreatment experiences. This might increase the risk to respond harshly to infant crying, possibly in an attempt to end the aversive signal [18,35,43]. Future research may consider examining whether individual differences in emotion regulation, impulsivity or personality traits might explain the association between fathers’ own childhood experiences and the ability to modulate handgrip force during infant crying.

We hypothesized that elevated HCC and HTC might underlie the association between experienced maltreatment and handgrip strength modulation. However, results revealed no associations of HCC and HTC with either experienced maltreatment or handgrip strength modulation. HCC and HTC did not mediate the association between childhood maltreatment and handgrip strength modulation. Regarding the link between childhood experiences and HCC, a meta-analysis on the effect of experienced adverse life events, including maltreatment, on HCC revealed an overall positive and significant association, albeit with a small effect size of *d* = 0.22 (CI 0.01, 0.43), for nonclinical samples that included adolescent or adult males (based on [28]). The current effect size for the association between experienced maltreatment and HCC was similar (β = 0.11, equivalent to *d* = 0.22), but non-significant, since our study was only powered to detect medium effect sizes. Studies including larger samples may be needed to further test whether childhood maltreatment relates to HCC in fathers, although it should be noted that based on our data a Bayes factor indicated more support for no association than for at least a small association between maltreatment and HCC. Importantly, previous studies differed in terms of strength and direction of the association between childhood maltreatment and HCC [28], suggesting that certain study characteristics may moderate the association. For example, severity of maltreatment experiences and timing of maltreatment could be potential moderators. Childhood experiences may be more strongly related to cortisol levels in samples that include individuals with severe maltreatment experiences [9] or individuals who experienced maltreatment early in life [45].

Experienced childhood maltreatment also did not relate to fathers’ HTC levels. We tentatively hypothesized to find a positive association, as life history theories propose that experiencing harsh parenting relates to accelerated pubertal development which may reflect heightened levels of hormones such as testosterone (e.g., [8,12,20]). However, no previous research assessed whether potential testosterone elevations in early adolescence relate to heightened testosterone in adulthood. The current study thus presents a first test of this hypothesis and findings suggest that experiencing childhood maltreatment does not relate to higher HTC levels in adulthood in fathers. Nevertheless, future research may test this association in different samples, e.g., in men with more severe maltreatment experiences, or in women as effects of negative parenting on pubertal development generally seem more pronounced in women [8].

HCC and HTC were unrelated to the use of excessive handgrip force during infant cry. Although no previous studies assessed relations between HCC and HTC and handgrip strength modulation in response to infant signals, studies have suggested that other hormones affect handgrip strength modulation. For instance, administration of oxytocin led to less excessive handgrip force in women without harsh parenting experiences [5] and increased endogenous oxytocin through massage related to reduced handgrip force in men [40]. Moreover, in expectant fathers, administration of vasopressin elicited more excessive handgrip force while watching a picture of an unknown infant compared to while watching a picture of one's own infant [3]. Given functional connections between neuropeptides (oxytocin and vasopressin) and steroid hormones (cortisol, testosterone; e.g. [11]), we expected to also find effects of cortisol and testosterone on handgrip strength modulation. An absence of main effects may occur when the hormones interact in their effects on parenting, and one previous study reported an interaction effect of salivary cortisol and testosterone on paternal sensitivity [12]. Here we explored whether HCC and HTC interacted in their effect on handgrip strength modulation, but found no interaction effect.

Unlike most previous studies on cortisol and testosterone and measures of parenting, we assessed hormone levels in hair. One could question whether associations between parenting and cortisol and testosterone may be more pronounced when using salivary hormone measures, which assess acutely circulating hormone levels, compared to hormones assessed in hair, representing longer-term cumulative hormone secretion. Previous studies obtained salivary measures either immediately before, after, or during a caregiving task (e.g. [12,42]), or close in time to the caregiving assessment, i.e., in the following week [27] and one could argue that concurrent factors affecting both salivary hormones and parenting may partly account for reported associations. However, in some cases these salivary hormones also predicted caregiving months later (e.g., [12,42]). Of note, an exploratory two-group analysis revealed a positive association between HCC and handgrip strength modulation in the group of expectant fathers, suggesting that expectant fathers with higher HCC used more excessive handgrip force during infant crying. However, we cannot conclude that the association between HCC and handgrip strength modulation was stronger for expectant fathers than new fathers as effect sizes in the two groups were not significantly different.

### 4.1 Limitations

The current findings should be considered in light of this study's limitations. First, the assessment of fathers' experienced childhood maltreatment was based on retrospective self-reports obtained around the same time as the handgrip assessment. This limits our ability to assess predictive effects, as we cannot rule out the possibility that concurrent factors affected both fathers' reporting of their childhood experiences and their handgrip strength modulation. We did assess whether current depressive symptoms may have confounded the association between childhood maltreatment and handgrip strength modulation and this was not the case. Second, retrospective and prospective reports of childhood maltreatment do not agree well [6]. Nevertheless, it has been argued that retrospective reports are valuable as they may identify a wider range of maltreatment cases compared to prospective measures based on official records that may capture only more severe cases of maltreatment [6]. Third, we chose to combine different childhood maltreatment experiences (e.g., abuse and neglect) into one score to reduce the number of tests, but future studies may distinguish between abuse and neglect experiences as this may be relevant for associations with hormone levels [37] and handgrip strength modulation [13], despite high genetic correlations between various types of maltreatment [58]. Fourth, our sample consisted of generally low-risk fathers, which limits the generalizability of the current findings. Therefore, an important question for future research is whether findings replicate in other, e.g., more at-risk samples. Fifth, many fathers had missing values for hair segments 2 and 3, because of their short hair length. In our main analyses we therefore focused on cortisol and testosterone secretion in the last month, rather than even longer-term hormone secretion based on the 2 cm or 3 cm hair segments. Nevertheless, results replicated in a sensitivity mediation analysis that included latent HCC and HTC factors based on all three 1 cm hair segments. Moreover, correlations among the three segments were substantial for HCC values (*r*s > 0.75) and HTC values (*r*s > 0.55), which aligns with previous findings for HCC across four 1 cm segments [51]. Based on these findings we suggest that future research aiming to measure HCC and HTC in men may aim to include only 1 cm of hair. In case the researchers' interest lies in significantly longer-term hormone secretion, they could consider to notify participants well in advance and request them to let their hair grow, or to repeatedly cut 1-cm segments of hair across multiple months. Finally, we used the handgrip force task as an indicator for parenting behavior rather than assessing actual parenting behavior. Although the handgrip paradigm does seem a valid indicator for parenting behavior as maltreating mothers have more difficulty modulating handgrip force to infant crying than non-maltreating mothers [16], future studies may include different or additional indicators of fathers' responses to infant crying.

### 4.2 Conclusion

In conclusion, the current study provided further evidence supporting the association between experienced childhood maltreatment and reduced ability to modulate handgrip force while listening to infant cries. To our knowledge, the current study was the first to explore associations of HCC and HTC with childhood experiences and handgrip strength modulation in fathers, an understudied group in parenting research. Hereby, we aimed to provide benchmarks for future research into the hormonal mechanisms that may underlie the association between fathers’ own childhood experiences and their parenting. We did not find evidence to support the hypothesis that HCC and HTC levels mediate between childhood maltreatment and handgrip strength modulation. We found small positive effect sizes for the direct paths to and from HCC and a small negative effect size for the path from HTC to handgrip strength modulation, although these effects were not significant and Bayes factors indicated that there was more evidence for no effects than for small effects. Nevertheless, future studies may examine these associations in more diverse and larger samples, to see whether findings converge. Moreover, although the inclusion of multiple hormones is a strength of this study, other potentially relevant endocrine factors for the association between childhood experiences and parenting such as oxytocin [5] and vasopressin [3,54] should be explored in future research to gain a better understanding of hormonal factors associated with the etiology of parenting behavior. Such work will help illuminate the mechanisms via which harsh parenting behavior is transferred over generations, which is crucial in informing opportunities for interventions with the aim to ultimately diminish harsh parenting and its negative consequences.

## CRediT author statement

Martine Verhees: Conceptualization, Methodology, Formal analysis, Data Curation, Writing – Original Draft, Visualization; Marinus van IJzendoorn: Conceptualization, Methodology, Writing—Review & Editing, Funding acquisition; Kim Alyousefi-van Dijk: Investigation, Data curation, Visualization, Project administration, Writing – Review & Editing; Anna Lotz: Investigation, Project administration, Writing – Review & Editing; Noor de Waal: Investigation, Data curation, Project administration, Writing – Review & Editing; Marian Bakermans-Kranenburg: Conceptualization, Methodology, Writing – Review & Editing, Supervision, Funding acquisition.

## Funding

This work was supported by a 10.13039/501100000781European Research Council grant (AdG 669249, 2015) awarded to Marian Bakermans-Kranenburg and a Spinoza grant (Netherlands Organization for Scientific Research) awarded to Marinus van IJzendoorn.

## Data availability statement

The data that support the findings of this study are not publicly available but are available from the corresponding author on reasonable request.
