# Supplementary material for: Child maltreatment affects fathers’ response to infant crying, not mediated by cortisol or testosterone
Source: Compr Psychoneuroendocrinol. 2021 Aug 28;8:100083. doi: 10.1016/j.cpnec.2021.100083 (PMC9216419; doi:10.1016/j.cpnec.2021.100083)
Supplement: Multimedia component 1 [file mmc1.docx]

**Supplementary Materials**

**Supplementary Materials S1. Inclusion and exclusion criteria**

A priori stated exclusion criteria for the current study were: not cohabitating with the biological mother of the child, no mastery of the Dutch language, a current endocrine, psychiatric or neurological disorder, a cardiovascular disease, MRI contraindications (e.g., metallic foreign objects, current or past serious head injury), the use of medication potentially interfering with the endocrine system, current heavy drinking, regular use of soft drugs, use of hard drugs within the past three months. For expectant fathers, the pregnancy had to be uncomplicated, of a singleton, and in week 18-31 at time of inclusion. Fathers whose partners used alcohol, tobacco or drugs were excluded. For new fathers, infants had to be singletons, healthy, full-term (i.e., born after 37 weeks gestation) and 2-4 months old at time of inclusion. New fathers were excluded when they used a baby carrier for over five hours per week as they participated in a larger intervention study that involved the use of a baby carrier. Inclusion and exclusion criteria were checked by trained researchers during telephone interviews before participant enrollment in the study.

**Supplementary Materials S2. Assessment of hair covariates**

To allow controlling for potential hair covariates of hair cortisol concentration and hair testosterone concentration in the analyses, participants completed a questionnaire that comprised questions about hair treatment. Specifically, ‘how often did you wash your hair per week in the past months’ (recoded into less or more than 3 times a week); ‘when was the last time you washed your hair’ (recoded into washed in the 24h before sampling, yes/no); ‘is your hair currently dyed’ (yes/no); ‘how many times per week did you use hair products (e.g., hair gel) in the past months’ (recoded into less or more than 3 times a week); ‘when was the last time you used a hair product’ (recoded into used a product in the 24h before sampling, yes/no). Moreover, participants reported on their length and weight (used to calculate BMI), medication use, i.e., ‘did you use medication in the past 48h’ (yes/no), and their highest completed education (recoded into years of education following primary school, which was used as an indicator for socio-economic status). Hair color of the participant was noted down by a research assistant during the visit and recoded into black, brown, blond, red and grey. Participants’ race was coded by researchers based on videotapes of fathers and was defined as Caucasoid type or other. Astronomical season was based on the date of the research visit. Hair segments were weighed at Dresden Labservices GMBH, allowing us to control for hair mass. Outliers for continuous covariates were winsorized: two for age father (*z* > 3.29); two for education father (*z* < -3.29), and two for BMI father (*z* > 3.29). There was no variance for the covariate hair dyed (i.e., none of the participants had dyed their hair) and therefore this variable was not used in further analyses.
